# 3D Printing of Bacteriophage‐Loaded Hydrogels: Development of a Local and Long‐Lasting Delivery System

**DOI:** 10.1002/adhm.202503113

**Published:** 2025-10-13

**Authors:** Corina Vater, Gopala Krishna Mannala, Max von Witzleben, Richard Frank Richter, Nike Walter, Michael Gelinsky, Volker Alt, Anja Lode, Markus Rupp

**Affiliations:** ^1^ Centre for Translational Bone Joint and Soft Tissue Research University Hospital Carl Gustav Carus and Faculty of Medicine Technische Universität Dresden 01307 Dresden Germany; ^2^ University Center of Orthopedic Trauma and Plastic Surgery University Hospital Carl Gustav Carus Technische Universität Dresden 01307 Dresden Germany; ^3^ Department of Trauma Surgery University Hospital Regensburg 93053 Regensburg Germany; ^4^ Department of Trauma Hand and Reconstructive Surgery University Hospital Giessen 35385 Giessen Germany

**Keywords:** 3D extrusion‐printing, alginate, bacteriophages, implant‐associated infections, laponite, methylcellulose, *Staphylococcus aureus*

## Abstract

Multiple drug‐resistant bacteria are a growing life‐threatening problem and novel treatment strategies are urgently needed. One promising option is the use of lytic bacteriophages, viruses that infect and kill bacteria with high specificity. To efficiently utilize bacteriophage therapy for the treatment of implant‐associated infections, an effective strategy for the local, long‐lasting administration of bacteriophages at the site of infection is required. With the aim of developing a defined delivery system, this study investigates the feasibility of 3D extrusion printing of bacteriophages embedded in biomaterial inks by using a *Staphylococcus aureus*‐specific phage strain as model. It is demonstrated that a bacteriophage‐loaded hydrogel blend consisting of alginate and methylcellulose (AlgMC) can be printed with high shape fidelity. After cross‐linking, the hydrogel constructs release bacteriophages that maintain their activity against *S. aureus* over a period of 35 days when incubated in human‐plasma‐like medium (HPLM). The integration of the nanoclay Laponite into the AlgMC blend, known for its high binding capacity for biomolecules, does not further prolong the release under (near) physiological conditions in HPLM but may protect bacteriophages under nonphysiological conditions. In conclusion, bacteriophage‐loaded AlgMC inks fulfill the requirements for local bacteriophage therapy as they release active bacteriophages in a sustained manner.

## Graphical abstract

This research investigates the feasibility of 3D‐printing of a bacteriophage‐containing hydrogel made of alginate and methylcellulose. The printed hydrogels steadily release active bacteriophages for up to 35 days which is beneficial to treat implant‐associated infections. Although adding the nanoclay Laponite does not prolong the release under physiological conditions, it may be advantageous in harsher environments.

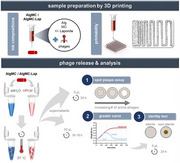

## 1 Introduction

Multiple drug‐resistant bacteria are a growing life‐threatening problem, they are thought to be responsible for more than 1.27 million deaths per year worldwide. Estimates predict an increase to 10 million deaths per year after 2050.^[^ ^1^ ^]^ In orthopedic and trauma surgery, implant‐associated infections such as periprosthetic joint infection and fracture‐related infection are devastating complications with high mortality rates.^[^ ^2^, ^3^, ^4^ ^]^ Limb reconstruction often requires a very long treatment period and amputation is often the last remaining surgical therapy option.^[^ ^5^, ^6^ ^]^ The number of implant‐associated infections is expected to rise in the coming decades, underlining the socioeconomic as well as the medical burden for the affected patients and healthcare systems.^[^ ^7^ ^]^ The emergence of antimicrobial resistance urgently requires novel treatment strategies and therefore, the World Health Organization endorses nontraditional approaches like bacteriophage therapy,^[^ ^4^ ^]^ whose potential was underlined in current reports.^[^ ^8^, ^9^ ^]^

Bacteriophages (phages) are viruses that infect bacteria and can be classified as being lytic or temperate. While lytic phages replicate in and finally lyse their bacterial host releasing new phages, temperate phages integrate into the genome of their host without killing it. Therefore, only lytic phages are used for phage therapy. Phages do not harm eukaryotic cells and have a narrow host range, enabling their selective use against pathogenic bacterial strains. Thus, phages are much more specific than antibiotics and do not affect the natural microbiome of a patient.^[^ ^10^, ^11^ ^]^ Potentially evolving phage resistance of bacteria can be overcome by using multiple phages (cocktails) and by exploiting the ability of phages to counteract resistance through coevolution.^[^ ^11^, ^12^ ^]^ Another advantage is that phages are self‐regulating, which enables an “adaptive therapy:” as long as bacteria are present at the site of infection, the phages replicate in them to infect more bacteria, and as soon as all bacteria are killed, the phages disappear. In clinical studies on implant‐associated infections, and in other medical disciplines, phage therapy has demonstrated promising results as antibacterial treatment in cases where standard treatments were ineffective.^[^ ^4^, ^13^, ^14^ ^]^

One of the unresolved questions is the optimal route of phage administration. In the treatment of implant‐associated infections, there is consensus that the local application of phages at the site of infection during or after surgical treatment is beneficial,^[^ ^15^ ^]^ and that an effective delivery strategy is pivotal for the clinical success.^[^ ^16^ ^]^ Therefore, much attention is currently being paid to the development and evaluation of biomaterial‐based delivery approaches. Depending on their nature, biomaterials can be loaded with phages by embedding (e.g., within hydrogels), encapsulation (e.g., into liposomes), and surface functionalization/coating (e.g., of ceramics or metals).^[^ ^17^ ^]^ Embedding/encapsulation can shield the phages against unfavorable environmental conditions in the human body, such as acidic pH, enzymatic attack, or serum inactivation, and thus, may prolong their half‐life in vivo. In addition, implantation of phage‐loaded biomaterials at the site of infection is expected to result in a sustained local release of the phages, which could maintain an antibacterial effect over a longer period of time without the need for repeated administrations,^[^ ^17^ ^]^ as is the case with phage suspensions administered through a draining system.^[^ ^18^, ^19^ ^]^

Hydrogels, consisting of biopolymers and/or synthetic polymers, are highly compatible with biological agents and flexible in their application, as they can be injected or implanted in preshaped form. To date, phage delivery has been investigated in a few in vitro and/or in vivo studies, mainly focusing on intestinal but also on the local application, for hydrogels based on alginate, chitosan, fibrin, agarose, and poloxamers.^[^ ^17^ ^]^ In two clinical cases of implant‐associated infections, phages were embedded in a defense antibacterial coating (DAC) hydrogel, available in the market, and the mixture was injected during surgery.^[^ ^20^, ^21^ ^]^ Both reports demonstrate the potential of localized phage therapy based on hydrogels. However, there is still an immense need for research to identify suitable hydrogel compositions and processing routes in order to achieve optimal delivery of active phages. The release kinetics of phages from a biomaterial matrix is defined by both its material properties and the implant design.^[^ ^17^ ^]^ Thus, while injection offers a high degree of flexibility in application, preshaped samples allow a better control.

3D printing technologies enable the precise shaping of hydrogels according to a predefined design in a computer‐aided additive manufacturing process. Using extrusion printing, the most common method in which the printing materials (“inks”) are strand‐wise deposited in layer‐by‐layer fashion, hydrogel constructs can be produced in clinically relevant dimensions.^[^ ^22^ ^]^ In addition to a defined outer size and shape, macropores can be integrated to facilitate exchange with the environment. Numerous hydrogel formulations have been developed for extrusion (bio)printing, with a strong focus on tissue engineering approaches by embedding mammalian cells.^[^ ^23^ ^]^ In recent years, the use of 3D (bio)printing has expanded to nonmalian cells,^[^ ^24^ ^]^ as well as complex active biomolecules such as enzymes, growth factors, and viral gene vectors for biotechnological applications and advanced delivery strategies.^[^ ^25^, ^26^, ^27^ ^]^

The present work explored the feasibility of 3D extrusion printing of phage‐loaded hydrogels with the aim of developing a defined delivery system that releases phages locally over a long period of time. With a blend of alginate and methylcellulose (AlgMC),^[^ ^28^, ^29^, ^30^, ^31^ ^]^ as well as a blend of AlgMC and the nanoclay Laponite (AlgMC–Lap),^[^ ^32^ ^]^ two ink formulations were used for phage printing that are established for bioprinting of mammalian cells and therefore well characterized. The alginate component forms the hydrogel network after ionic cross‐linking whereas the methylcellulose stabilizes the ink during the printing process due to its high viscosity; after printing and cross‐linking, the methylcellulose is released.^[^ ^28^, ^29^ ^]^ The integration of Laponite has been shown to enhance the binding capacity of the hydrogel for drugs, and growth factors;^[^ ^26^, ^32^, ^33^ ^]^ which can be attributed to the large and highly ionic surface area of the disk‐shaped nanoparticles that make up this synthetic magnesium silicate clay.^[^ ^34^, ^35^ ^]^

In this study, the hypothesis was tested that phage‐loaded hydrogel constructs can be fabricated with high shape fidelity by extrusion 3D printing and that active phages are released from these gels for a prolonged time by using a *Staphylococcus aureus*‐specific phage strain as model. It was investigated to what extent the phages integrated in the inks influence their printability and whether the addition of Laponite has an impact on the release of active phages.

## 2 Experimental Section

### 2.1 Bacteriophages

*S. aureus*‐specific phages (191219; D&D Pharma GmbH, Pyrmont, Germany) were propagated either in liquid culture or via the double layer agar method using the *S. aureus* strain EDCC 5055 (DSM No. 28763; DSMZ, Braunschweig, Germany) as described previously.^[^ ^36^ ^]^ In brief, for propagation in liquid culture, *S. aureus* bacteria were grown in Lysogeny Broth (LB) medium at 37 °C until an OD_600_ of 1 and then infected with the phages by addition of 5 mL phage suspension (5 × 10^8^ plaque forming units (PFU) mL^−1^) to 25 mL bacterial suspension. After overnight incubation, phages were separated from bacteria and debris by centrifugation (10 min at 5086 × *g*); the supernatant was filtered through 0.45 and 0.2 µm filters. To obtain high phage titers, the double layer agar technique was used for propagation of phages. Therefore, 100 µL of *S. aureus* grown in tryptone soy broth (TSB) medium with an OD_600_ of 0.2 was mixed with 100 µL of phages (1 × 10^6^ PFU mL^−1^) and incubated at room temperature for 10 min. After mixing with 3 mL of warm liquid 0.5% TSB soft agar, the mixture was poured over 20 mL solid 1.5% TSB agar in a petri dish and after agar polymerization incubated statically over night at 37 °C. The next day, 6 mL of SM phage buffer (100 mm NaCl, 8 mm MgSO_4_, 50 mm TRIS‐HCl, pH 7.5) with 1 mm CaCl_2_ was added per dish and dishes were incubated at room temperature on an orbital shaker at 80 rpm. After 4 h, buffer and soft agar were transferred into 50 mL tubes and centrifuged (10 min at 4415 × *g*) to sediment agar and bacterial debris. The remaining supernatant was filtered through 0.45 and 0.2 µm filters. Phage titers were determined by using the spot plaque assay as described.^[^ ^37^ ^]^

### 2.2 Printing Materials

#### 2.2.1 Ink Preparation

Alginic acid sodium salt (Alg; β‐d‐mannuronate (M)/α‐l‐guluronate (G) ratio 1:2, Sigma‐Aldrich, Germany), methylcellulose (MC; 4000 cP, Sigma‐Aldrich, USA), and Laponite (Lap; Laponite XLG, BYK Additives & Instruments, UK) were sterilized as powders by autoclaving at 121 °C for 20 min. AlgMC inks were prepared by dissolving 3 wt% Alg and 9 wt% MC in either phosphate buffered saline (PBS) or directly in phage suspension (1 × 10^6^, 1 × 10^9^, 2 × 10^9^, or 1 × 10^11^ PFU mL^−1^ in TSB). For preparation of AlgMC–Lap inks, 3 wt% Lap was dissolved first in PBS or phage suspension and then, 3 wt% Alg and 6 wt% MC were added. The inks prepared with PBS as solvent were loaded with phages by addition of 100 µL phage suspension (2 × 10^9^ PFU mL^−1^) to 1 mL ink. Phage‐free inks using TSB as solvent were prepared for printing the negative controls.

#### 2.2.2 Rheological Characterization

To determine the viscosity of the inks with different phage concentrations (1 × 10^9^, and 1 × 10^11^ PFU mL^−1^; phage‐free inks in TSB or PBS as negative controls), rotational measurements with a plate–plate rheometer (MCR 301, Anton Paar, Austria; plate diameter: 50 mm, plate distance: 0.1 mm) were conducted (*n* = 3). The shear rate was continuously increased up to 100 s^−1^ within 20 min. All inks were investigated shortly after mixing and after storing them at room temperature for 24 h.

### 2.3 3D Printing

The extrusion‐printing system BioScaffolder 3.1 (GeSiM mbH, Radeberg, Germany), operated under sterile conditions at room temperature, was used for 3D printing of defined hydrogel samples. The inks were filled into cartridges (Globaco GmbH, Roedermark, Germany) and dispensed through conical needles (Nordson EFD GmbH, Germany) with different outlet diameters (200–840 µm) as indicated for each experiment.

#### 2.3.1 Sample Preparation for the Long‐Term Release Experiments

Samples consisting of a single strand with a length of 5 mm were printed using 840 µm needles, an applied pressure of 40–45 kPa, and a printing speed of 8 mm s^−1^. After printing, the samples were incubated in 100 mm CaCl_2_ solution for 10 min to cross‐link the alginate chains.

#### 2.3.2 Filament Fusion and Filament Collapse Test

In order to characterize the shape fidelity of phage‐loaded inks during printing, filament fusion and filament collapse tests for all rheologically characterized AlgMC compositions were carried out according to Ribeiro et al.^[^ ^38^ ^]^ Both tests were performed with 410 µm needles and a printing speed of 10 mm s^−1^ whereas applied air pressure ranged 70–80 kPa. Three layers of strands, connected by meanders, were printed on top of each other with increasing strand distance. The resulting structures were imaged with the stereo light microscope (Olympus SZX16); the strand widths, strand distances, and fused segment lengths were measured using ImageJ (1.51w, National Institutes of Health, USA). Dividing the fused segment length (*f*_s_) by the strand width (*f*_d_) gave the shape fidelity ratio, whose optimum was set at 1. The correlation between the shape fidelity ratio and the respective strand distance provided information about the printing accuracy at a certain strand distance. For the filament collapse test determining the shape fidelity of a strand covering two strands of certain distance from a horizontal perspective, a strand of ink was deposited on a polylactic‐acid‐based device simulating different strand distances of 1.0, 2.0, 4.0, 8.0, and 16.0 mm of the underlying layer. The quality of the bridging could be assessed by measuring how horizontal the ink bridges the gaps, the more horizontal the ink, the lesser the ink was prone to fail the bridging. Therefore, the angle between the platforms and the free hanging ink was measured, with a horizontal line equal to 90°.

#### 2.3.3 Proof of Principle Experiment

Bulk and macroporous scaffolds were printed with 410 µm needles, an applied pressure of 75–85 kPa, and a printing speed of 10 mm s^−1^. Bulk scaffolds were designed with a radius of 4 mm and a height of 4.2 mm. They were printed with 23 layers, each rotated by 90° relative to the previous one; the strands were placed without distance. The macroporous scaffolds were printed with the same volume of ink as the bulk scaffolds. Therefore, they were printed with a radius of 8 mm and a height of 4.2 mm (23 layers). The macropores were created by placing strands 3.5 mm apart. Each layer was rotated by 90° relative to the previous one, resulting in a grid‐like pore architecture. After printing, the samples were incubated in 100 mm CaCl_2_ solution for 30 min including a turning of the scaffolds inside the well plates after 15 min to ensure a homogenous cross‐linking from all sides.

### 2.4 Antibacterial Activity

#### 2.4.1 Release

The printed samples were incubated either in ultrapure water (Milli‐Q) or human‐plasma‐like medium (HPLM; Gibco, FisherScientific, USA) at 37 °C. In experiments without change of the release solution, single‐strand samples were incubated in 5 mL and every day, 300 µL was collected for analysis. In experiments with change of the release solution, single‐strand samples were incubated in 1 mL and scaffolds in 5 mL; at different time points of incubation, release solutions were completely replaced with fresh medium and the collected eluates were analyzed.

#### 2.4.2 Activity against Planktonic Cultures

Growth curve analyses were carried out as follows: fresh *S. aureus* overnight cultures in LB medium were adjusted to an OD_600_ of 0.4. 50 µL of the eluates were added to 100 µL of this bacterial suspension and the OD_600_ of these cultures was monitored over an incubation period of 16 h at 37 °C (measurement every 10 min). After 16 h, a sterility test was carried out by spotting 5 µL of the cultures into each well of a 12‐well plate filled with 2% solid LB agar. After overnight incubation at 37 °C, wells were checked for growing colonies to determine bacterial survival in comparison to the positive control (sterile or not sterile). For the spot plaque assay, 100 µL of *S. aureus* cultures in LB medium with an OD_600_ of 0.4 were mixed with 3 mL warm liquid 0.5% LB soft agar following dispensing the mixture at 200 µL per well into 12‐well plates already filled with 2% solid LB agar (1 mL per well). After agar polymerization, 5 µL of the release solutions were spotted per well onto the LB soft agar, followed by overnight incubation at 37 °C. Plaques were documented microscopically and analyzed semiquantitatively by applying a score ranging from 0 (no plaques at all) to 4 (complete lysis).

#### 2.4.3 Biofilm Formation Inhibition Assay

The potential of released phages to inhibit the formation of biofilms was tested by incubating 100 µL of supernatant or control with 100 µL of fresh *S. aureus* overnight cultures in LB medium with an OD_600_ of 1 statically at 37 °C for 48 h. Samples were then washed 3× with ddH_2_O, fixed by adding 200 µL per well ice‐cold methanol, and incubated at RT for 10 min. After removal of the methanol, samples were air‐dried, stained for 5 min at RT with 200 µL per well 0.1% crystal violet, carefully washed 3× with ddH_2_O, and air‐dried again. For quantitative analysis of biofilm formation, solubilization of the crystal violet was carried out by adding 200 µL per well 33% acetic acid followed by incubation at RT for 15 min on an orbital shaker at 450 rpm and measurement of the absorbance at 570 nm.

### 2.5 pH Measurement

For measuring the pH, frozen eluates from samples incubated in ultrapure water were thawed overnight at 4 °C, equilibrated to room temperature for 10 min, and vortexed before starting the measurement. Measurement of the pH value was carried out in 1.5 mL tubes containing the supernatants using a Sentix Mic‐D electrode (FisherScientific).

### 2.6 Ion Quantification

To quantify the amount of calcium, magnesium, sodium, lithium, and silicon ions in the supernatants of AlgMC and AlgMC–Lap samples incubated in water, inductively coupled plasma‐optical emission spectroscopy (Plasma Quant PQ 90 0 0 Elite, Analytik Jena, Jena, Germany) was used. Therefore, 0.5 mL of the supernatant was diluted 1:10 and acidified with 2% nitric acid. The following wavelengths were used for emission intensity measurements: 315.887 nm (calcium), 279.078 nm (magnesium), 330.237 nm (sodium), 670.791 nm (lithium), and 251.611 nm (silicon).

### 2.7 Statistical Analysis

All values – except the ones for PFU scores – were evaluated by two‐way analysis of variance, followed by Tukey's multiple comparison test. For statistical analysis of PFU scores (categorical values), data were transferred into frequency tables following the nonparametric Fisher's exact test. Statistical analyses for all data were performed using GraphPad Prism 10.2.3 software and significant differences were assumed at *p* < 0.05.

## 3 Results

### 3.1 Activity against Planktonic *S. aureus* Cultures

#### 3.1.1 3D Printed Alginate‐Based Hydrogels Release Active Phages

Two loading procedures were compared: i) similar to preparation of cell‐laden bioinks,^[^ ^28^ ^]^ the AlgMC blend was first prepared by dissolving the powder components in PBS and then, the phage suspension was mixed in at a ratio of 1:10 immediately before printing (“AlgMC in PBS + phages”); ii) the powder components were directly dissolved in the phage suspension, resulting in an ≈10 times higher phage amount in the ink (“AlgMC in phage suspension”). Both compositions demonstrated excellent printability, independent from the phage concentration.

The first release experiment should answer the fundamental question whether active phages are released from the printed and cross‐linked hydrogels. Single‐strand samples produced from both variants were incubated in water over 4 days without refreshing the eluent. Growth curve analysis revealed a significant antibacterial activity of the release solutions taken from “AlgMC in phage suspension” (≈2 × 10^9^ PFU mL^−1^ in the ink) every day. The longer the incubation period, the earlier the onset of reduction in bacterial density was observed, which can be attributed to an accumulation of the released phages over time (Figure S1A, Supporting Information). For the release solutions taken from “AlgMC in PBS + phages” (≈2 × 10^8^ PFU mL^−1^ in the ink), a much lower antibacterial activity, indicated by a significant later drop of the growth curves, was detected which might be a result of the lower amount of loaded phages (Figure S1A, Supporting Information). In a repeat experiment, similar differences between both variants were observed (Figure S2, Supporting Information). Results of the plaque forming assay confirmed these observations: compared to the “AlgMC in PBS + phages” group, a higher density of plaques, indicating bacterial clearance, was visible in the “AlgMC in phage suspension” group which also increased with incubation time (Figure S1B, Supporting Information).

#### 3.1.2 Laponite Enhances the Activity of Phages Released in Water and Prolongs Their Delivery

The Laponite‐containing inks were loaded in the same way by either i) mixing the phage suspension into the AlgMC–Lap blend prepared beforehand in PBS (“AlgMC–Lap in PBS + phages” with 2 × 10^8^ PFU mL^−1^), or ii) by dissolving the powder components directly in the phage suspension (“AlgMC–Lap in phage suspension” with 2 × 10^9^ PFU mL^−1^). The resulting phage‐loaded inks were printable as well.

During incubation of printed and cross‐linked single‐strand samples for 4 days in water without refreshing, the release solutions, collected from both variants every day, showed a significant antibacterial activity, as indicated by a decrease in bacterial density in the growth curve analysis as well as by plaque formation (Figure S1, Supporting Information). Again, a higher activity of the release solutions collected from the “AlgMC–Lap in phage suspension” in comparison to those of the “AlgMC–Lap in PBS + phages” group was observed, albeit the difference was less pronounced. Compared to its Laponite‐free counterpart, “AlgMC–Lap in PBS + phages” showed a higher activity (Figure S1, Supporting Information), indicating that Laponite‐supported hydrogels are advantageous to enhance the antibacterial efficacy of phages released in water, especially at lower amounts of loaded phages. The repeat experiment confirmed these observations (Figure S2, Supporting Information).

**Figure 1:**
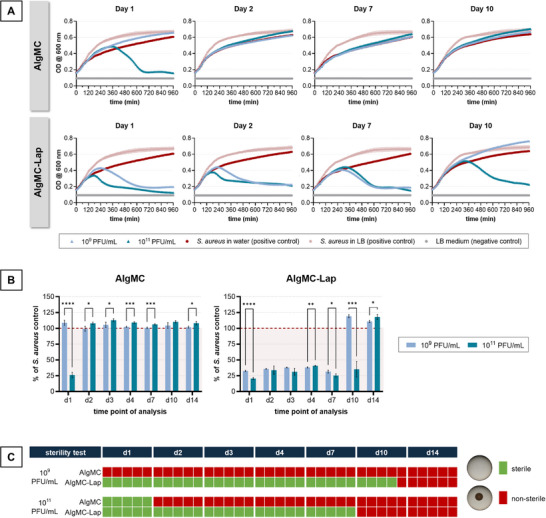
Antibacterial activity of phages released from printed AlgMC and AlgMC–Lap hydrogels: growth curve analysis with change of the eluent at each time point of analysis. The inks were prepared by dissolving the biomaterials in phage suspensions of either 1 × 10^9^ or 1 × 10^11^ PFU mL^−1^. Release was examined by incubating the samples in water at 37 °C. A) Growth curves of *S. aureus* in presence of release solutions for selected time points; in addition to the standard *S. aureus* positive control cultivated in LB medium, another *S. aureus* positive control was included, in which the release solution was replaced by water (mean ± standard deviation (SD); *n* = 5). B) Growth curve end point measurement (16 h) for the release solutions related to the positive control with water (mean ± SD; *n* = 5; **** *p* < 0.0001, *** *p* < 0.001, ** *p* < 0.01, * *p* < 0.05). C) Sterility spot assay after 16 h of incubation in presence of release solutions; sterile: no growing bacterial colonies, nonsterile: growing bacterial colonies, either isolated or completely merged (*n* = 5).

The release from “AlgMC versus AlgMC–Lap in phage suspension” was studied over 14 days with regular refreshing of the eluent to answer the question how long active phages are released from the printed and cross‐linked hydrogels, and whether the presence of Laponite prolongs the delivery. The inks were prepared by dissolving the powder components in phage suspensions of either 1 × 10^9^ or 1 × 10^11^ PFU mL^−1^. **Figure** **1**A shows the growth curves for selected time points and Figure 1B the optical density after 16 h (growth curves end point measurement) for all time points. While release solutions of AlgMC showed antibacterial activity only on day 1 and only if loaded with the higher amount of phages (1 × 10^11^ PFU mL^−1^), release solutions effective against *S. aureus* could be collected from AlgMC–Lap until day 7 (1 × 10^9^ PFU mL^−1^ group) and day 10 (1 × 10^11^ PFU mL^−1^ group) (see also Figure S3 in the Supporting Information for statistical analysis regarding the material effect). An antibacterial effect of the biomaterials was excluded by testing AlgMC and AlgMC–Lap samples without phage loading (Figure S4, Supporting Information). After growth curve analysis, the cultures were subjected to a sterility spot assay to detect any remaining live bacteria. In the presence of all release solutions which caused a drop in the growth curve, all bacteria were lysed, indicated by the absence of growing colonies (Figure 1C).

**Figure 2:**
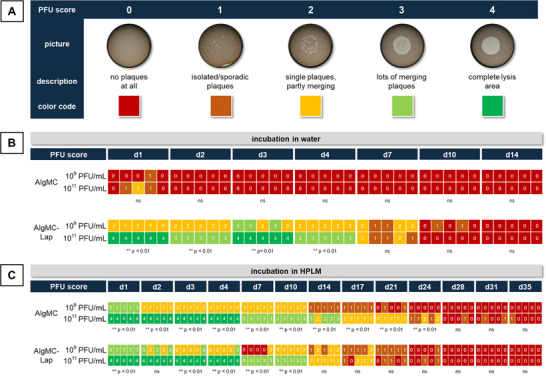
Antibacterial activity of phages released from printed AlgMC and AlgMC–Lap hydrogels: PFU spot assay with change of the eluent at each time point of analysis. The inks were prepared by dissolving the biomaterials in phage suspensions of either 1 × 10^9^ or 1 × 10^11^ PFU mL^−1^. A) Score defined in the study to assess plaque formation intensity as a measure for phage activity. Assessment of PFU formation according to the score for the AlgMC and AlgMC–Lap groups after B) incubation in water or C) HPLM at 37 °C. For statistical analysis, scoring data were transferred into frequency tables following Fisher's exact test (performed day‐ and material‐wise) to evaluate the influence of phage concentration (10^9^ PFU mL^−1^ vs 10^11^ PFU mL^−1^; *α* = 0.05, *n* = 5, ** *p* < 0.01, ns = not significant).

Plaque formation was tested semiquantitatively in a PFU spot assay; for data evaluation, a score was defined (**Figure** **2**A). The data confirmed the findings of the growth curve analysis: for AlgMC loaded with 1 × 10^9^ or 1 × 10^11^ PFU mL^−1^, no or only isolated plaques were detected on day 1 (Figure 2B). With AlgMC–Lap, high scores (3–4, indicating merging plaques or completely lysed area) were determined for the 1 × 10^11^ PFU mL^−1^ group and medium scores (2, indicating partially merging plaques) for the 1 × 10^9^ PFU mL^−1^ group within the first 4 days. Low scores (1–2, indicating single/isolated plaques) were determined for both groups (1 × 10^9^ and 1 × 10^11^ PFU mL^−1^) on day 7 whereas nearly no plaques were visible in both groups on days 10 and 14.

#### 3.1.3 The Release of Active Phages Strongly Depends on the Surrounding Conditions

**Figure 3:**
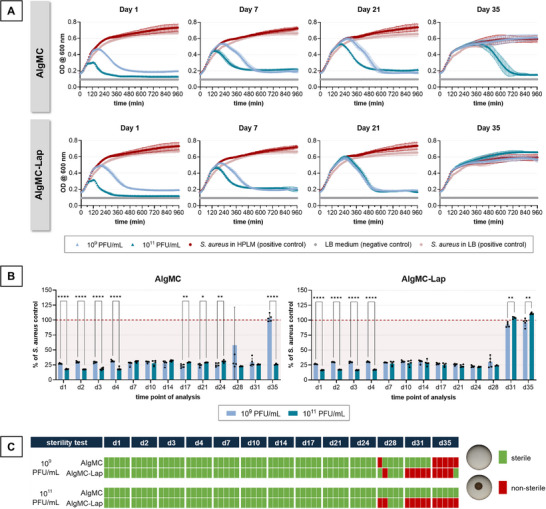
Antibacterial activity of phages released from printed AlgMC and AlgMC–Lap hydrogels: growth curve analysis with change of the eluent at each time point of analysis. The inks were prepared by dissolving the biomaterials in phage suspensions of either 1 × 10^9^ or 1 × 10^11^ PFU mL^−1^. Release was examined by incubating the samples in HPLM at 37 °C. A) Growth curves of *S. aureus* in presence of release solutions for selected time points; in addition to the standard *S. aureus* positive control cultivated in LB medium, another positive control was included, in which the release solution was replaced by HPLM (mean ± SD; *n* = 5). B) Growth curve end point measurement (16 h) of release solutions related to the positive control with HPLM (mean ± SD; *n* = 5, **** *p* < 0.001, ** *p* < 0.01, * *p* < 0.05). C) Sterility spot assay after 16 h of incubation in presence of release solutions; sterile: no growing bacterial colonies, nonsterile: growing bacterial colonies, either isolated or completely merged (*n* = 5).

In parallel, a release experiment was carried out using HPLM^[^ ^39^ ^]^ as eluent to simulate the natural environment in the human body. Compared to water, a much longer release of active phages and a generally higher activity was observed in HPLM while a positive effect of Laponite was not observed. Growth curve analysis revealed that the release solutions obtained from AlgMC exhibited activity against *S. aureus* up to the collection time points of day 31 (1 × 10^9^ PFU mL^−1^ group) and day 35 (1 × 10^11^ PFU mL^−1^ group) (**Figure** **3**A,B). The sterility spot assay confirmed that the activity of the released phages was sufficient to lyse all bacteria until the mentioned time points, since no growing bacterial colony was observed (Figure 3C). Release solutions from both AlgMC–Lap groups (1 × 10^9^ and 1 × 10^11^ PFU mL^−1^) showed a reliable antibacterial effect up to day 24. On day 28, some samples were not releasing phages with sufficient activity, as indicated by a high standard deviation in the growth curve endpoint measurements as well as by partially unsterile samples in the sterility spot assay (Figure 3B,C).

The PFU spot assay revealed a concentration dependence of the antibacterial activity of the release solutions, as indicated by higher scores for the groups (AlgMC and AlgMC–Lap) loaded with 1 × 10^11^ PFU mL^−1^ compared to those loaded with 1 × 10^9^ PFU mL^−1^ (Figure 2C). In addition, the score decreased over time in all groups: for groups loaded with 1 × 10^9^ PFU mL^−1^, the score was between 1 and 2 (AlgMC–Lap) or fell below 2 (AlgMC) after 10 days. For groups loaded with 1 × 10^11^ PFU mL^−1^, scores equal or below 2 were detected after 14 (AlgMC–Lap) and 21 days (AlgMC) (Figure 2C).

#### 3.1.4 Laponite Stabilizes the pH Value and Increases the Ion Concentration

**Figure 4:**
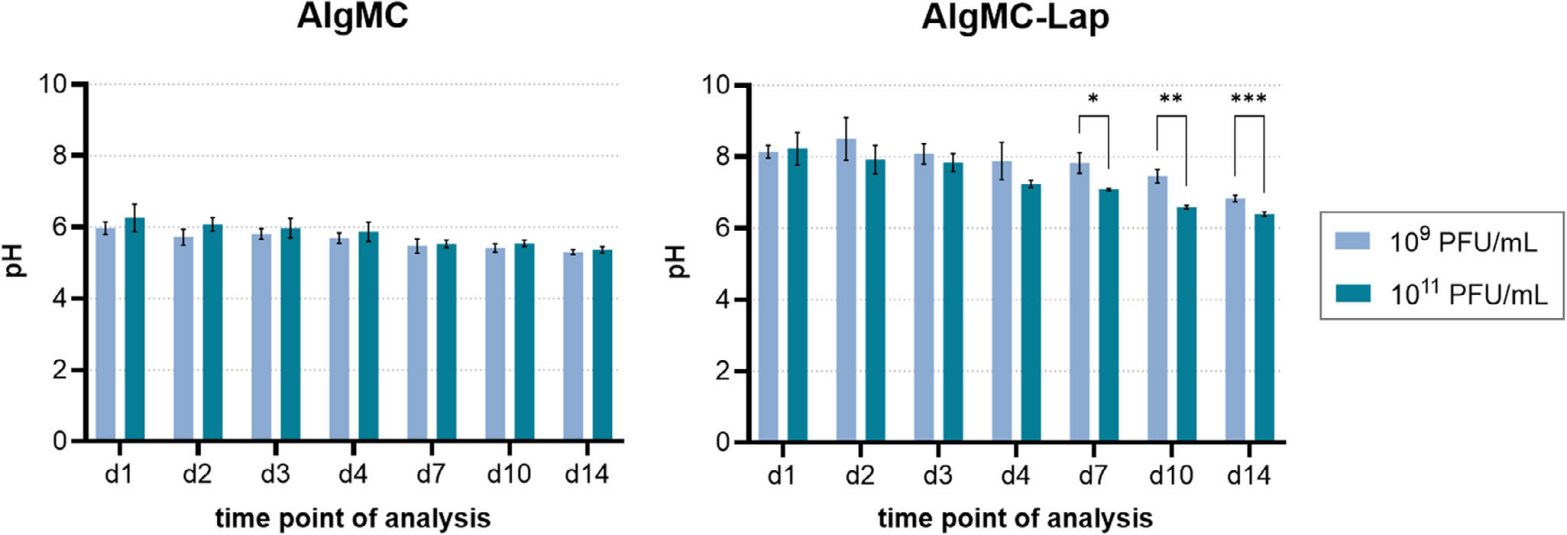
Measurement of the pH value in release solutions collected from printed AlgMC and AlgMC–Lap hydrogels (change of the eluent at each time point of analysis). The inks were prepared by dissolving the biomaterials in phage suspensions of either 1 × 10^9^ or 1 × 10^11^ PFU mL^−1^. Samples were incubated in water at 37 °C (mean ± SD; *n* = 5, *** *p* < 0.001, ** *p* < 0.01, * *p* < 0.05).

To explore the reason for the positive effect of Laponite on the release of active phages during incubation in water, the pH value and the concentration of ions, that can theoretically be released from the biomaterials, were determined in the release solutions. The pH value was significantly influenced by the presence of Laponite in the hydrogels (**Figure** **4**): within the first days (days 1–3), pH values around 6 were measured in the supernatants of AlgMC samples, while the pH value in the supernatants of AlgMC–Lap was around 8. Later on, the pH decreased continuously in all supernatants; on day 14, values of around 5.5 and 7 were measured for AlgMC and AlgMC–Lap, respectively. Loading of the hydrogels with phages had no effect on the pH in case of AlgMC, but slightly, nonsignificantly higher values were observed in the AlgMC–Lap group loaded with 1 × 10^9^ PFU mL^−1^ (Figure S5, Supporting Information).

**Figure 5:**
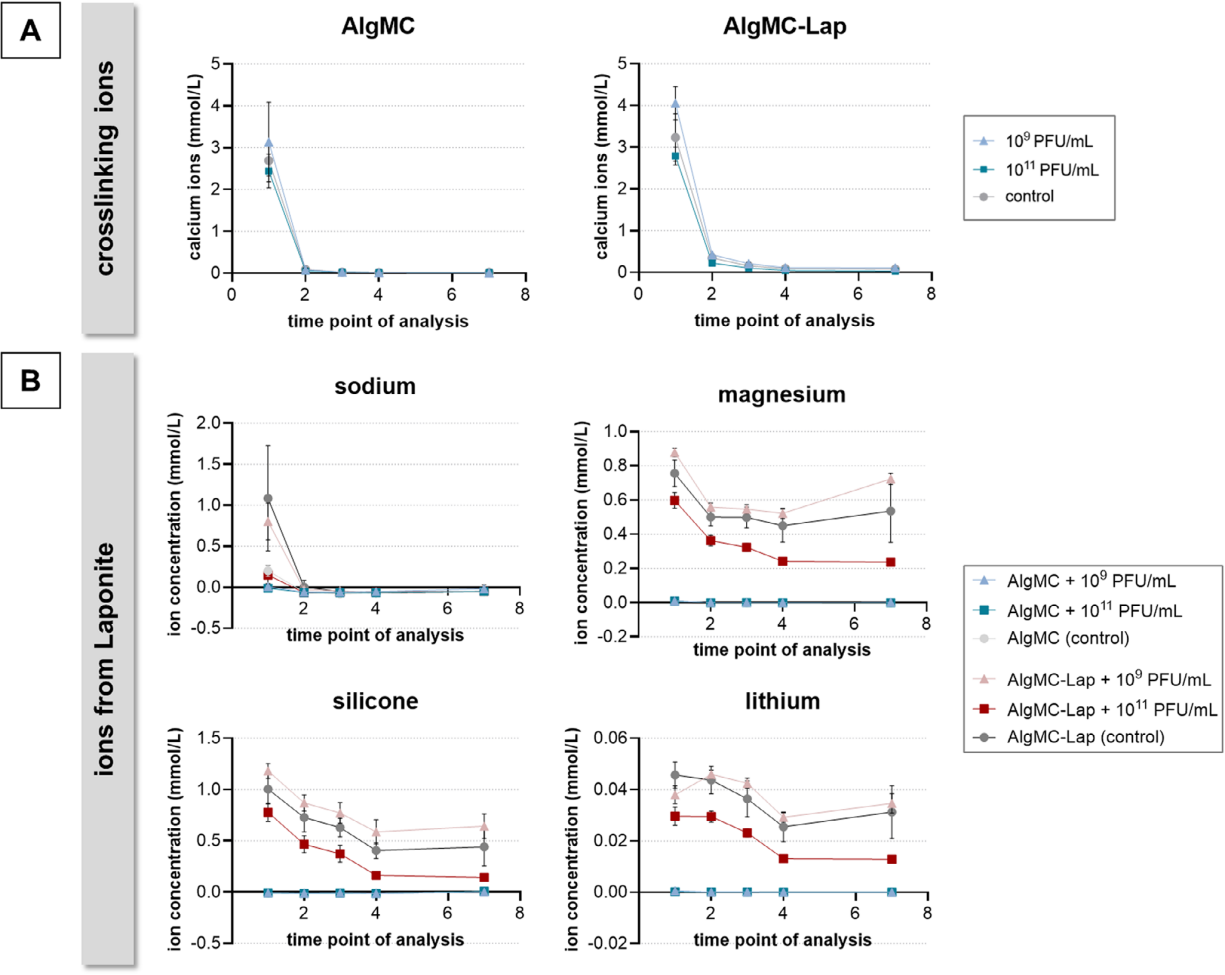
Quantification of ions in release solutions collected from printed AlgMC and AlgMC–Lap hydrogels (change of the eluent at each time point of analysis). The inks were prepared by dissolving the biomaterials in phage suspensions of either 1 × 10^9^ or 1 × 10^11^ PFU mL^−1^ or in TSB medium (control). Samples were incubated in water at 37 °C (measured ion concentration in water blanks are already subtracted, mean ± SD; *n* = 5): A) release of the cross‐linking calcium ions and B) release of ions from Laponite. For comparison, ion concentrations in HPLM were 3.34 mmol L^−1^ for calcium, 141.57 mmol L^−1^ for sodium, 1.23 mmol L^−1^ for magnesium, 0.03 mmol L^−1^ for silicone, and 0.001 mmol L^−1^ for lithium.

After printing, the alginate chains in the hydrogels were cross‐linked by incubation in 100 mm CaCl_2_ solution. A small proportion of the cross‐linking calcium ions were released during incubation of the samples in water (**Figure** **5**A): on day 1, concentrations between 2.4 and 3.1 mmol L^−1^ (AlgMC groups) and between 2.7 and 4 mmol L^−1^ (AlgMC–Lap groups) were measured. After day 1, only marginal amounts of calcium ions, below 0.4 mmol L^−1^, were measured in all groups.

The release of sodium, magnesium, silicon, and lithium ions from the AlgMC–Lap samples is indicative of Laponite degradation.^[^ ^35^ ^]^ Sodium ions were released only on day 1 from the AlgMC–Lap and the nonloaded AlgMC samples (Figure 5B). A constant release of magnesium ions was observed from the hydrogels containing Laponite, with the AlgMC–Lap group loaded with 1 × 10^9^ PFU mL^−1^ phages having the highest concentration and the AlgMC–Lap group loaded with 1 × 10^11^ PFU mL^−1^ having the lowest concentration of magnesium ions in the supernatants (Figure 5B). The release of silicon ions from AlgMC–Lap samples was in the range of 0.7–1.2 mmol L^−1^ on day 1 and decreased over time to 0.1–0.6 mmol L^−1^ on day 7. Lithium ions were only detected in very low concentrations, below 0.05 mmol L^−1^, in the release solutions collected from AlgMC–Lap samples. Again, the group loaded with 1 × 10^9^ PFU mL^−1^ phages released more silicon and lithium ions than the group loaded with 1 × 10^11^ PFU mL^−1^ phages. As expected, magnesium, silicon, and lithium ions were not detected in the release solutions obtained from AlgMC samples.

### 3.2 Printing 3D Constructs from Phage‐Loaded AlgMC Inks

Since the AlgMC‐derived hydrogel showed more favorable results in terms of the long‐term release of active phages, it was analyzed in more detail with regard to the influence of loaded phages on printing properties.

#### 3.2.1 Phage Suspensions as Solvent of the AlgMC Ink Influence its Viscosity

**Figure 6:**
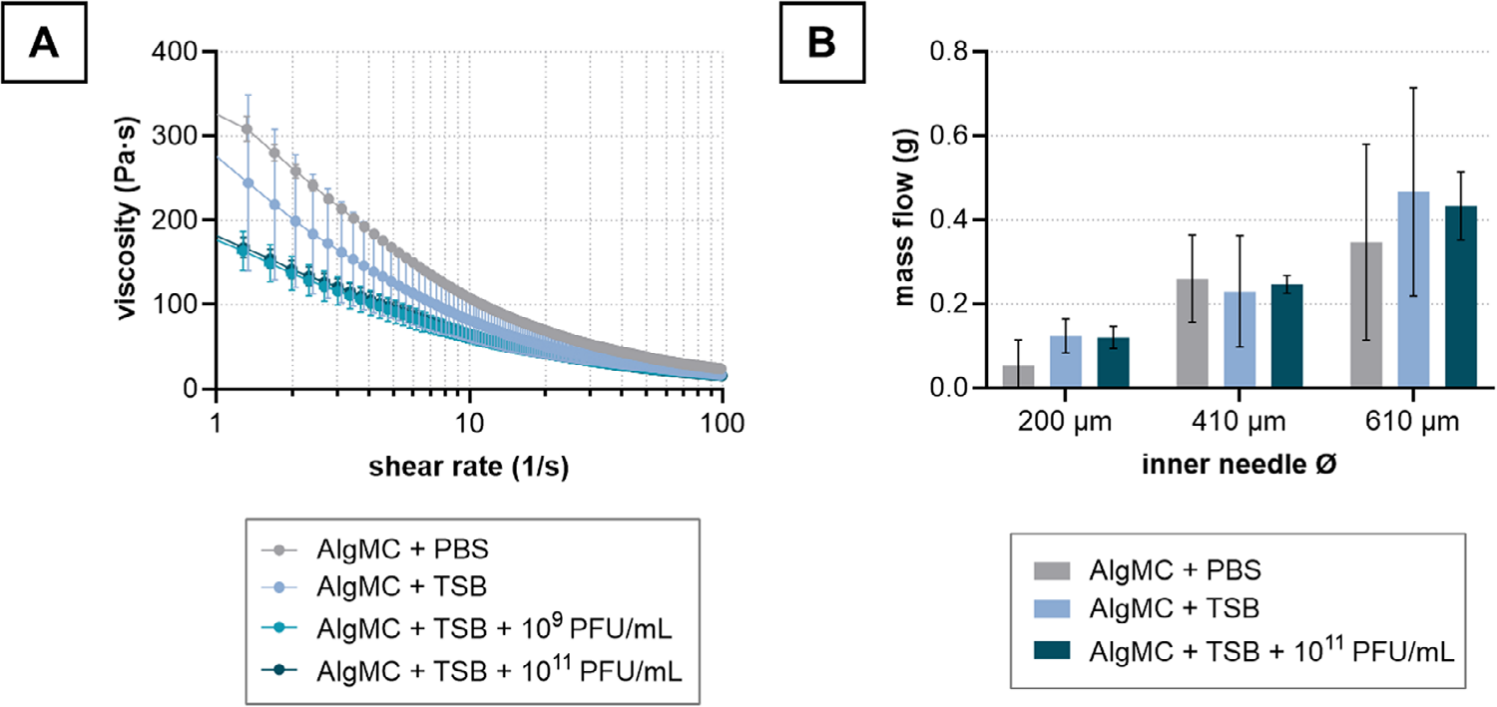
Printing 3D constructs from phage‐loaded AlgMC inks. A) Rheological properties and B) mass flow were assessed. The inks were prepared by dissolving the alginate and methylcellulose in phage suspensions of either 1 × 10^9^ or 1 × 10^11^ PFU mL^−1^ or in phage‐free TSB medium or PBS (mean ± SD; *n* = 5).

AlgMC with 1 × 10^9^ PFU mL^−1^ and AlgMC with 1 × 10^11^ PFU mL^−1^ were rheologically characterized in comparison to AlgMC in TSB w/o phages as well as AlgMC in PBS. For all printing materials, a beneficial shear‐thinning behavior was determined whereas the viscosity of the AlgMC ink was reduced when PBS as solvent was replaced by TSB (**Figure** **6**A). The presence of different phage concentrations in TSB tended to decrease the viscosity further, but without a significant difference between the two phage concentrations. The mass flow during extrusion is influenced by the needle diameter (as shown in Figure S6 in the Supporting Information for the “AlgMC in TSB” ink), as well as by the viscosity. A lower viscosity can lead to a higher mass flow when extruding material that in turn influences its printing properties. However, when all material compositions were extruded with 100 kPa for 30 s, a similar mass flow was observed through needles of the same inner diameter (200, 410, 610 µm); the mass flow increased with increasing needle diameter (Figure 6B).

#### 3.2.2 Phage Suspensions as Component of the AlgMC Ink Slightly Affect the Shape Fidelity

**Figure 7:**
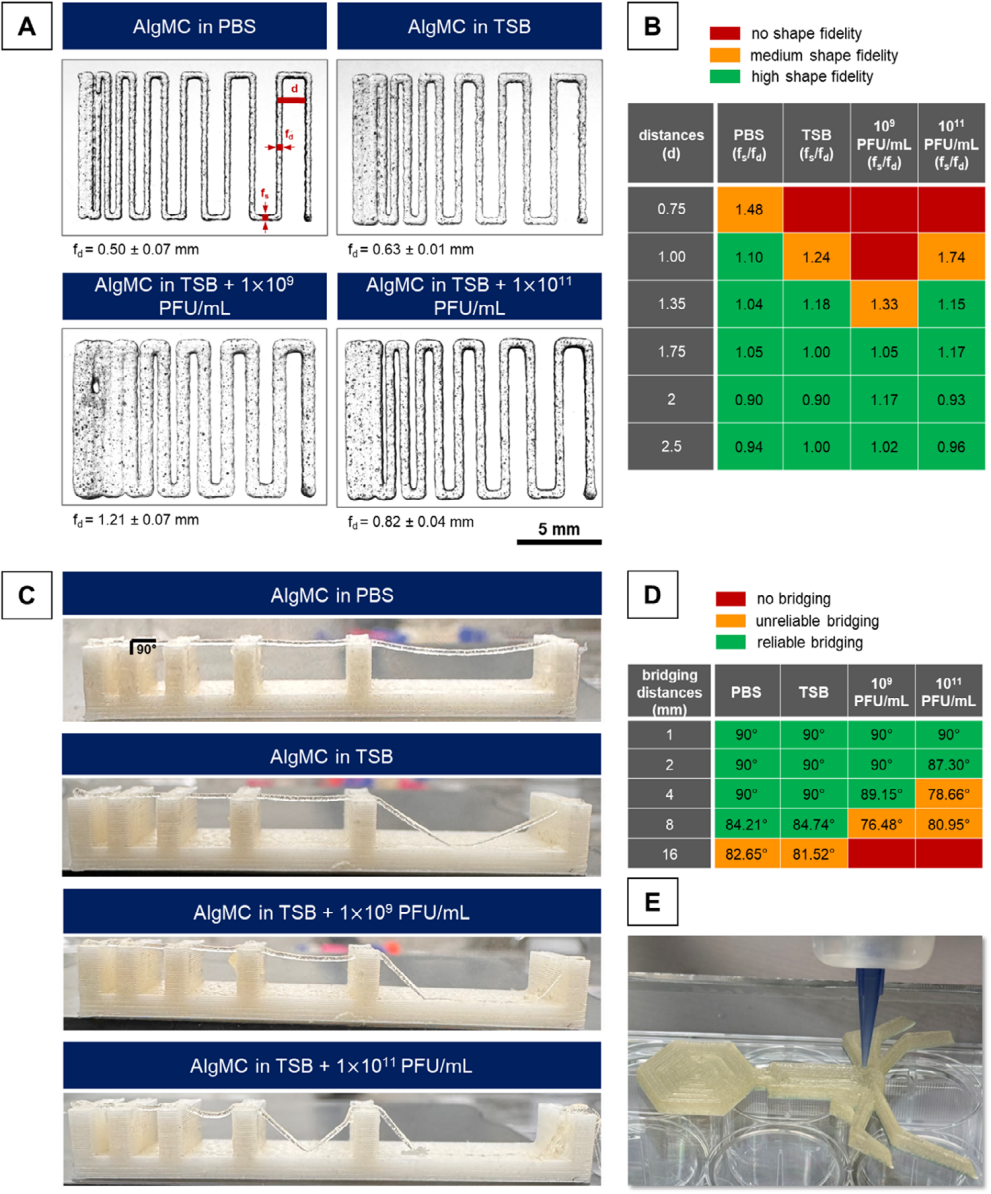
The printability was characterized by investing the shape fidelity of printed strands with A,B) filament fusion and C,D) filament collapse tests. The inks were prepared by dissolving the alginate and methylcellulose in phage suspensions of either 1 × 10^9^ or 1 × 10^11^ PFU mL^−1^ or in phage‐free TSB medium or PBS; printing was carried out through a 410 µm needle (*f*_s_: fused segment length, *f*_d_: strand width). E) Printing was carried out with AlgMC in TSB + 1 × 10^11^ PFU mL^−1^ phages through a 410 µm needle (50 layers).

Filament collapse and filament fusion tests are proven methods to quantify the shape‐fidelity of 3D printed constructs,^[^ ^38^ ^]^ and were used to describe the printing properties of AlgMC loaded with phages (1 × 10^9^ and 1 × 10^11^ PFU mL^−1^) in comparison to AlgMC in TSB w/o phages and AlgMC in PBS (**Figure** **7**). The lowered viscosity increased the strand diameter leading to a fusion of strands for small strand distances (Figure 7A). Therefore, no shape fidelity was maintained when printing pores with strand distances below 1 mm upon the use of TSB medium or phage suspensions as ink component. But as soon as the strand distances increased (≧1 mm), the division *f*_s_/*f*_d_ showed an overall high shape fidelity independent from the material composition (Figure 7B). On the other hand, the lowered viscosity of the AlgMC inks prepared with TSB medium or phage suspensions decreased the bridging capacity, especially for higher concentrations of phages (Figure 7C). AlgMC dissolved in PBS or TSB was able to bridge the biggest gap of 16 mm in most cases. However, no bridging occurred after the addition of phages, whereas the concentration of 10^11^ PFU mL^−1^ resulted in a more unreliable bridging even for the smaller gaps (Figure 7D). Despite the somewhat reduced shape fidelity, the phage‐loaded AlgMC inks are applicable for reliable 3D printing of predesigned constructs (Figure 7E).

#### 3.2.3 Proof‐of‐Principle: Printing of Phage‐Loaded Constructs According to a Predefined Design

**Figure 8:**
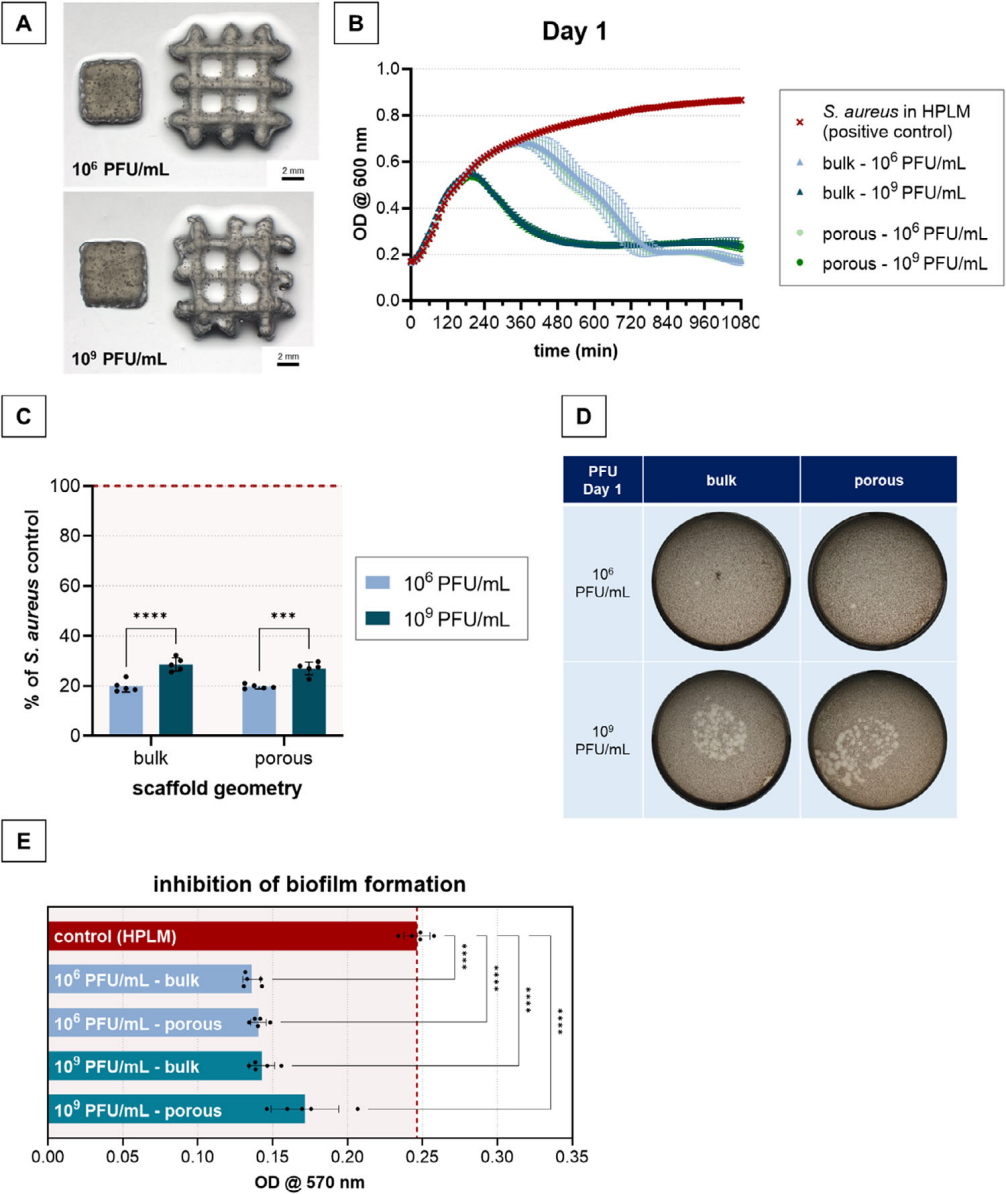
3D printing of phage‐loaded AlgMC scaffolds according to a predefined design and testing the antibacterial activity of released phages. The inks were prepared by dissolving the biomaterials in phage suspensions of either 1 × 10^6^ or 1 × 10^9^ PFU mL^−1^; printing was carried out through a needle with an inner diameter of 410 µm. Release was examined by incubating the samples in HPLM at 37 °C for 24 h. A) Printed and cross‐linked scaffolds with bulk and macroporous geometry. B) Growth curves of *S. aureus* in presence of release solutions and C) growth curve end point measurement (18 h) for the release solutions related to the *S. aureus* positive control (release solution replaced by HPLM; mean ± SD, *n* = 5; **** *p* < 0.0001, *** *p* < 0.001). D) Spot plaque assay and E) biofilm inhibition assay. Therefore, release solutions were incubated together with *S. aureus* for 48 h before staining the formed biofilm with crystal violet following quantitative analysis by solubilizing the crystal violet and measuring the optical density (mean ± SD, *n* = 5, **** *p* < 0.0001).

AlgMC loaded with 1 × 10^6^ and 1 × 10^9^ PFU mL^−1^ were printed as bulk and macroporous scaffolds (**Figure** **8**A). The cross‐linked samples were incubated in HPLM and the antibacterial activity of release solutions collected after 24 h was investigated by growth curve analysis and spot plaque assay (Figure 8B–D). Both scaffold types – bulk and macroporous – released active phages. While a significantly earlier onset of bacterial density reduction was observed for the scaffolds loaded with 1 × 10^9^ PFU mL^−1^, even with the low – more clinically relevant – loading amount of 1 × 10^6^ PFU mL^−1^, all bacteria were lysed after the 18 h incubation period of the growth curve, as indicated by the sterility spot assay. Additionally, it was proven that the released phages were able to prevent the formation of biofilms: compared to the positive control (release solution replaced by HPLM), the presence of all release solutions significantly inhibited biofilm formation (Figure 8E). In contrast to the growth curve analysis and spot plaque assay, inhibition of biofilm formation was independent from the amount of phages that were loaded into the scaffolds.

## 4 Discussion

This study demonstrated the compatibility of 3D extrusion printing of alginate‐based hydrogel inks with bacteriophages, enabling the creation of long‐term delivery systems for phages that are defined in shape and size. This could not only enable better control of phage–hydrogel application in terms of amount and distribution in comparison to current clinical procedures, but also allow for the prefabrication of ready‐to‐use patches or fleeces, which would reduce the intraoperative workload for surgeons by eliminating the need to prepare phage‐loaded hydrogels on‐site. In addition, our work provides a basis for the integration of phages into bioprinted constructs – either in vivo implants or in vitro tissue models – to prevent implant‐associated infections or contaminations in in vitro‐testing systems.

In this study, on the one hand, the phages maintained their activity when embedded in the hydrogels due to the mild process conditions (physiological temperature and pH, avoidance of organic solvents) and the ink compositions which have been shown in previous studies to be suitable for bioprinting of living cells.^[^ ^28^, ^30^, ^31^, ^32^ ^]^ On the other hand, although a higher number of phages tended to decrease the viscosity and shape fidelity of the inks, an adequate printing quality was maintained. The data also showed that both – the composition of the biomaterial and the environmental conditions during release – strongly influenced the duration of the release of active phages. Specifically, this study provided valuable insights into the general suitability and limitations of using Laponite as delivery component, which is described as beneficial for, e.g., anticancer drugs and growth factors such as BMP‐2,^[^ ^40^, ^41^ ^]^ for bacteriophages.

To ensure a sustained, long‐lasting delivery of phages, the hydrogel matrix should have the capacity to retain the phages and release them slowly over time. Phages have an average size of around 50–200 nm and consist of a protein‐based capsid containing nucleic acids and mostly have a tail ending in a base plate with fibers, also composed of proteins. Therefore, phages are large biomolecule complexes which contain positively charged (tail) and negatively charged (head) domains.^[^ ^42^ ^]^ Retention of biomolecules in a hydrogel matrix is based on noncovalent bonds between the biomolecules and the biomaterial, which can be formed by ionic interactions, hydrogen bonds, van der Waals forces, and hydrophobic interactions.^[^ ^43^ ^]^ The release rate of embedded biomolecules depends on the adsorption/desorption kinetics as well as their diffusion within the hydrogel, which is the predominant mechanism to transport a biomolecule in this environment.^[^ ^44^ ^]^ Depending on the pore size and the size of the biomolecule (complex), a hydrogel matrix can also act as physical barrier, significantly slowing the diffusion of a biomolecule and thus, influencing the release rate.

After printing and ionic cross‐linking of AlgMC, the resulting hydrogel constructs consisted mainly of a Ca^2+^–alginate network since the methylcellulose molecules are released.^[^ ^28^, ^29^ ^]^ Alginate is a linear polysaccharide composed of M and G residues, with one carboxyl group on each residue and therefore, a negative net charge above pH 4.^[^ ^45^ ^]^ The carboxyl groups can electrostatically interact with divalent cations such as Ca^2+^, forming gels, but also with positively charged segments of biomolecules.^[^ ^43^, ^45^ ^]^ Therefore, ions and biomolecules compete for binding; many proteins exhibited a low encapsulation efficiency and fast release from alginate gels.^[^ ^43^, ^46^ ^]^ The smaller the size of a biomolecule, the faster the diffusion through the nanoporous alginate network.^[^ ^46^, ^47^ ^]^ Compared to proteins, phages are about 10–50 times larger, suggesting physical entrapment and slow diffusion as the main mechanisms for their sustained release from AlgMC hydrogels.

Physical entrapment and release are dependent on the pore size and cross‐linking density of an alginate gel matrix which in turn is influenced by the conditions during gel formation and the release experiment. In the AlgMC system, ionic cross‐linking of the alginate chains took place in the presence of methylcellulose macromolecules. In a previous study, we have demonstrated by scanning electron microscopy analysis of dried samples that the resulting matrix is less dense and has a microporous surface compared to cross‐linked samples of pure alginate.^[^ ^28^ ^]^ This altered micro‐/nanostructure indicates a (partially) increased mesh size of the alginate network that could be beneficial for the release of the large bacteriophages.

Laponite is a layered magnesium lithium silicate nanoclay that crystalizes into disk‐shaped nanoparticles with a diameter and thickness of 25–30 and 1 nm, respectively.^[^ ^34^ ^]^ The nanodisks have a strong negative charge on their surface and at pH values < 9, a weak positive charge at the edge; the net negative charge is balanced by cations such as Na^+^, which are located between the nanodisks.^[^ ^34^, ^35^, ^48^ ^]^ Laponite has a high cation exchange capacity and therefore, by releasing Na^+^ in aqueous media, (cationic) biomolecules can be bound via electrostatic interactions.^[^ ^35^ ^]^ When dispersed in aqueous solutions at appropriate Laponite concentrations and ion strength, the nanodisks self‐assemble into an ordered configuration (often referred as to “house‐of‐cards” structure) by preferential interactions between the negatively charged disk surfaces and the positively charged edges, forming a weak and shear‐thinning gel.^[^ ^35^, ^48^ ^]^ Integrated biomolecules can not only interact with the nanoparticle surfaces and edges but also intercalate into the space between the nanoparticles, so that in addition to noncovalent bonds, barrier effects delaying diffusion can also be involved in the retention of a biomolecule.^[^ ^34^ ^]^ The release of the biomolecules occurs through an ion‐exchange mechanism and via diffusion through the inter‐nanoparticle space.^[^ ^35^ ^]^ Laponite has been investigated as drug delivery system, alone and in combination with polymers, for small molecules such as DOX and other anticancer drugs,^[^ ^35^ ^]^ as well as for larger molecules such as the proteins BMP‐2,^[^ ^49^ ^]^ and VEGF;^[^ ^50^ ^]^ its biocompatibility has been proven in many studies.

Here, Laponite was integrated into the AlgMC system to exploit its high retention capacity for biomolecules to further prolong the release of phages. In this complex system, interactions between the single components of the phage‐loaded ink – the polymer macromolecules, the Laponite nanodisks, (cat)ions, and the phages – occur. This may specifically alter the Laponite nanodisk organization – smaller units of assembled nanodisks can be assumed – and may also influence the alginate network formation. Scanning electron microscopy analysis in our previous study showed the formation of a heterogeneous matrix of AlgMC–Lap with porous and compact areas, while the surface was generally dense and smooth.^[^ ^32^ ^]^ Nevertheless, in the present study, stable gels were obtained after incubation in CaCl_2_ solution, which maintained their stability throughout the incubation period. Therefore, the embedded phages were expected to be slowed down by both physical entrapment and strongly delayed diffusion, as well as strong binding to the Laponite nanodisks by electrostatic interactions.

The release of the phages from the hydrogels was first analyzed in deionized water. Although nonphysiological, this extreme condition helps to understand the impact of the individual biomaterial components. In water, AlgMC‐derived hydrogel samples only released phages with sufficient antibacterial activity if they were loaded with a higher number of phages and only for a short time. When incubated without change of the eluent, phages accumulated in the release solutions (Figure 1; Figures S1 and S2, Supporting Information), indicating that they were continuously released in this 4 days period and that they maintained their activity at least partially after the release. However, when incubated with daily change of the eluent, only on day 1 an antibacterial activity in the release solutions was determined (Figures 1 and 2B). This could be explained by either an insufficient number of released phages at later time points or a strong reduction of the activity of released phages.

Looking at the release of phages from the AlgMC‐derived samples in HPLM, which mimics physiological conditions, insufficient release after day 1 seems unlikely since the release extracts in HPLM showed an antibacterial activity until day 31/35 (10^9^/10^11^ PFU mL^−1^ group; Figures 2C and 3). A fast degradation of the alginate network in water, which could also lead to an initial burst release, is unlikely, as by measuring calcium in the supernatant – an indicator of weakening of the Ca^2+^–alginate network – an appreciable concentration of ≤3 mm was only detected on day 1, but this is far below the concentration of the cross‐linking solution (100 mm). Consistently, the AlgMC‐derived samples were stable in water over a period of 35 days. Therefore, it is more likely that the phages are not sufficiently stable and have lost their activity: After being released in water, they were exposed to adverse conditions since the pH value in the release solutions was ≤6 and nearly no ions were present (Figures 4 and 5).

Phages are much more complex and therefore more labile compounds than other antimicrobials and have been described to be unstable outside a certain range of environmental conditions (temperature, pH, salt concentrations).^[^ ^11^ ^]^ For the phage strain used in the present study, the impact of different pH values in the range between 2 and 12 was investigated (Figure S7, Supporting Information): at pH ≤ 6, the activity was significantly reduced compared to pH = 7.5 (the reference value of the SM‐phage buffer) as indicated by growth curve analysis and spot plaque assay. In comparison to AlgMC, the pH value in the release solutions collected from AlgMC–Lap was with 7–8 significantly higher (Figure 4) and phage activity was detected until day 7 (10^9^ and 10^11^ PFU mL^−1^ groups) and partially until day 10 (10^11^ PFU mL^−1^ group), even with regular refreshment of the eluent (Figures 1 and 2B).

Another possible reason for this loss of phage activity could be damage of the phage capsid, as observed by Furiga et al.^[^ ^51^ ^]^ While dilution of MS2 phages in ultrapure water (extremely low ionic strength) led to a loss of capsid structural integrity and reduction of infectivity by breakup of the capsid, phages in PBS (high ionic strength) kept their activity and no capsid breakage was detected. The phage stabilizing effect of alkali and alkaline earth cations like lithium, sodium, potassium, and magnesium was demonstrated as early as 1953 by Lark and Adams.^[^ ^52^ ^]^ They found that these ions could prevent killing of phages when exposed to high temperatures of 50–70 °C and concluded, based on physicochemical kinetics and electron microscopy, that ionic environment might affect the substructure of phage virion. Later, these observations were confirmed by Bourdin et al. who observed that in the presence of 10 mm magnesium ions, T4 phages showed no loss of titer over 1 month when stored at 30 °C.^[^ ^53^ ^]^ Strongly related to the presence of ions in the phages’ surrounding environment, another possible reason for a decreased phage activity could be phage aggregation. Langlet et al. demonstrated that a pH below the isoelectric point of the specific phage led to phage aggregation with sizes of few micrometers that in turn resulted in a strong decrease of PFU counts.^[^ ^54^ ^]^ A novel mechanism of aggregation/disaggregation transitions by phage virions discovered by Szermer‐Olearnik et al. confirmed these findings.^[^ ^55^ ^]^ While low ionic strength led to phage aggregation of 20–100 virions, return to high ionic strength reversed this process. Using sodium as an example, they showed that lowering the Na^+^ concentration below 20 mm causes rapid phage aggregation while returning Na^+^ concentration to normal sodium levels in phage media (150 mm) causes dispersion of phages. Their results suggest an ion‐mediated kind of quorum sensing among phages where loss of ionic strength may act as a trigger in an evolutionary mechanism to improve phage survival by stimulating phage aggregation when outside a bacterial host. This mechanism might also explain the findings in our study. When incubated in ultrapure water, ionic degradation products of the Laponite nanoparticles (Si(OH), Mg^2+^, and Li^+^) were continuously released from AlgMC–Lap samples (Figure 5), as it is known for Laponite in aqueous environments at pH below its isoelectric point (pH ≈10).^[^ ^35^, ^40^ ^]^ This resulted in an increased ionic strength and pH value in the surrounding environment that might have stabilized the phages and decreased phage aggregation leading to a higher phage activity.

It can be assumed that the AlgMC–Lap samples released less phages per time point in water than the AlgMC samples due to the higher retention capacity of the hydrogels containing Laponite, but that this lower amount of phages was much better stabilized regarding their activity, which ultimately led to a longer‐lasting antibacterial effect. In the high‐ionic strength system of HPLM, the effect of ions released from Laponite (Figure 5B) is negligible leading to an even shorter release of active phages from AlgMC–Lap (until day 24) compared to AlgMC (until day 31/35) (Figures 2C and  3). Thus, under (nearly) physiological conditions, the integration of Laponite into the AlgMC hydrogel is rather disadvantageous, as too little phages seemed to be released due to an excessive retention.

To our knowledge, the use of nanoclays as a component of a phage delivery system has not yet been reported. Alginate‐based hydrogels have been investigated in other studies,^[^ ^56^, ^57^, ^58^, ^59^, ^60^, ^61^, ^62^ ^]^ however, the comparison of our phage release data with those from the literature is difficult due to the large heterogeneity of experimental setups (different phage strains, loaded amounts, sample sizes, release conditions, and activity tests). Nevertheless, a sustained delivery of active phages from alginate hydrogels was also described by others. Ismail et al. found a prolonged release time of two weeks in LB medium and a faster bacterial lysis kinetics of phages (*Escherichia coli*‐specific λvir) released from β‐tricalcium phosphate (β‐TCP) bone substitute materials coated with 1% alginate containing the phages compared to β‐TCP directly coated with the phages.^[^ ^56^ ^]^ Stipniece et al. assumed a sustained release of *S. aureus*‐specific phages from alginate matrices, as only 0.3% of the loaded amount was released in TRIS‐HCl buffer (pH 7.4) after 72 h.^[^ ^58^ ^]^ By contrast, Barros et al. observed at physiological pH in PBS that 40% of phages (*Enterococcus faecalis*‐specific LM99) were already released after 30 min from 2% alginate hydrogels with and without NanoHAp, followed by 97% release after 24 h.^[^ ^57^ ^]^ To our best knowledge, there is only one other study that investigated 3D printed alginate hydrogels with embedded phages: Shen et al. reported that 10% of the phages targeting H5a *E. coli* were released in 24 h and that the antibacterial effect lasted for at least 24 h.^[^ ^63^ ^]^ A sustained release of active phages was also reported for other hydrogels, for example, fibrin glue (*Pseudomonas aeruginosa*‐specific PA5 phages, release in 0.9% NaCl over 11 days),^[^ ^64^ ^]^ and poloxamer P407 (*E. faecalis*‐specific EFDG1 and EFLK1 phages, release in PBS over 28 days).^[^ ^65^ ^]^

Some studies investigated the efficacy of hydrogel‐based phage delivery systems in in vivo infection models. Cobb et al. observed in a rat model that *S. aureus*‐specific phages delivered by injected alginate gels were successful in reducing soft tissue but not bone infections.^[^ ^59^ ^]^ Wroe et al. engineered an injectable hydrogel based on poly(ethylene glycol)‐4‐maleimide for delivery of phages targeting *P. aeruginosa* and achieved a nearly fivefold reduction in live *P. aeruginosa* counts at the infection site at day 7 postimplantation in an infected murine bone defect model.^[^ ^66^ ^]^ Onsea et al. tested a phage delivery system based on an emulsion‐based hydrogel (a mixture of soy phospholipids, soybean oil, glycerin, and water, added to carboxymethylcellulose) in a clinically relevant rabbit model of fracture‐related infections caused by *S. aureus* in comparison to the application of the phages in saline through a subcutaneous access tube.^[^ ^67^ ^]^ Besides providing a proof of concept regarding the application of phage therapy for such infections, this study also demonstrated that the delivery of the phages via a hydrogel minimizes the risk of superinfection through the access tube and limits the exposure of the phages to the adaptive immune system, indicated by the absence of phage neutralization in the hydrogel group.^[^ ^67^ ^]^ Shafigh Kheljan et al. investigated the antimicrobial effect of alginate–carboxymethylcellulose hydrogels loaded with either *P. aeruginosa*‐specific phages or the antibiotic ciprofloxacin in a mouse wound infection model. They found a similar antimicrobial effect of phage‐ and antibiotic‐containing hydrogels, but a better outcome for the phage‐containing hydrogels in terms of wound healing.^[^ ^62^ ^]^

When 3D printing hydrogel constructs according to a predefined design, shape fidelity is an important aspect which in turn is influenced by the rheological properties of the ink. In addition to the biomaterials themselves, the solvent of the powder components also has a considerable impact on these properties. In this work, phage loading of the ink by direct dissolving the biomaterial powders in the phage suspensions, i.e., TSB medium containing the phages, allows for the integration of the highest possible amount of phages in the ink. However, the TSB medium already instead of PBS as solvent had a visible effect on viscosity and shape fidelity (Figure 7). In addition to salts (like in PBS), TSB medium contains also sugars, amino acids, casein, and (poly)peptides – in other words, molecules that can interact with the ink components and therefore change the intrinsic properties of the ink. Similarly, the use of fresh frozen human blood plasma as solvent, that also contains proteins, results in a decrease in viscosity compared to AlgMC dissolved in PBS.^[^ ^68^ ^]^ The additional presence of phages in the ink further increased the density of the biomolecules with charged domains and a correspondingly greater change in viscosity and print shape fidelity was observed (Figures 7 and 8). However, these changes did not affect the printability of constructs according to a predefined design, as they can be largely compensated for by adjusting the printing parameters. In future research – toward clinical application – purified phage suspensions and potentially also lower phage numbers will be used; that in turn will also result in a reduced effect of the phage suspension on the rheological and printing properties of a hydrogel.

## 5 Conclusion

In order to efficiently utilize bacteriophage therapy for the treatment of severe implant‐associated infections, an effective strategy for the local, long‐lasting administration of phages at the site of infection is required. Hydrogels are promising candidates for local phage delivery systems due to their high compatibility with biological agents and flexibility in application. In this study, a hydrogel blend of alginate and methylcellulose, suitable for extrusion 3D printing with high shape fidelity, was shown to release active phages over a period of five weeks. The hydrogel matrix is able to effectively retain the embedded phages and protect them over this period. The data also show that the phages can survive in the hydrogel in the absence of their bacterial host, which would allow the application of this phage delivery system also for prevention of infections. The phage‐loaded hydrogels can be 3D printed with high shape fidelity according to a predefined design while maintaining the activity of the embedded phages. The integration of the nanoclay Laponite, which should further prolong the release time, has not been shown to be beneficial under (near) physiological release conditions in HPLM, as too strong binding of the phages prevents the release of a sufficient number of active phages at later time points, but may be beneficial under conditions where the pH is lowered, e.g., during inflammation/infection.

## Conflict of Interest

The authors declare no conflict of interest.

## Supporting information



Supporting Information

## Data Availability

The data that support the findings of this study are available from the corresponding author upon reasonable request.
