# Supplementary material for: 3D Printing of Bacteriophage‐Loaded Hydrogels: Development of a Local and Long‐Lasting Delivery System
Source: Adv Healthc Mater. 2025 Oct 13;15(5):e03113. doi: 10.1002/adhm.202503113 (PMC12864588; doi:10.1002/adhm.202503113)
Supplement: Supplementary file 1 — Supporting Information [file ADHM-15-0-s001.pdf]

ADVANCED HEALTHCARE MATERIALS

Supporting Information

for *Adv. Healthcare Mater.*, DOI 10.1002/adhm.202503113

3D Printing of Bacteriophage-Loaded Hydrogels: Development of a Local and Long-Lasting Delivery System

Corina Vater, Gopala Krishna Mannala, Max von Witzleben, Richard Frank Richter, Nike Walter, Michael Gelinsky, Volker Alt, Anja Lode and Markus Rupp**

Supporting Information

3D Printing of Bacteriophage-loaded Hydrogels: Development of a Local and Long-lasting Delivery System

Corina Vater, Gopala Krishna Mannala, Max von Witzleben, Richard Frank Richter, Nike Walter, Michael Gelinsky, Volker Alt, Anja Lode, Markus Rupp**

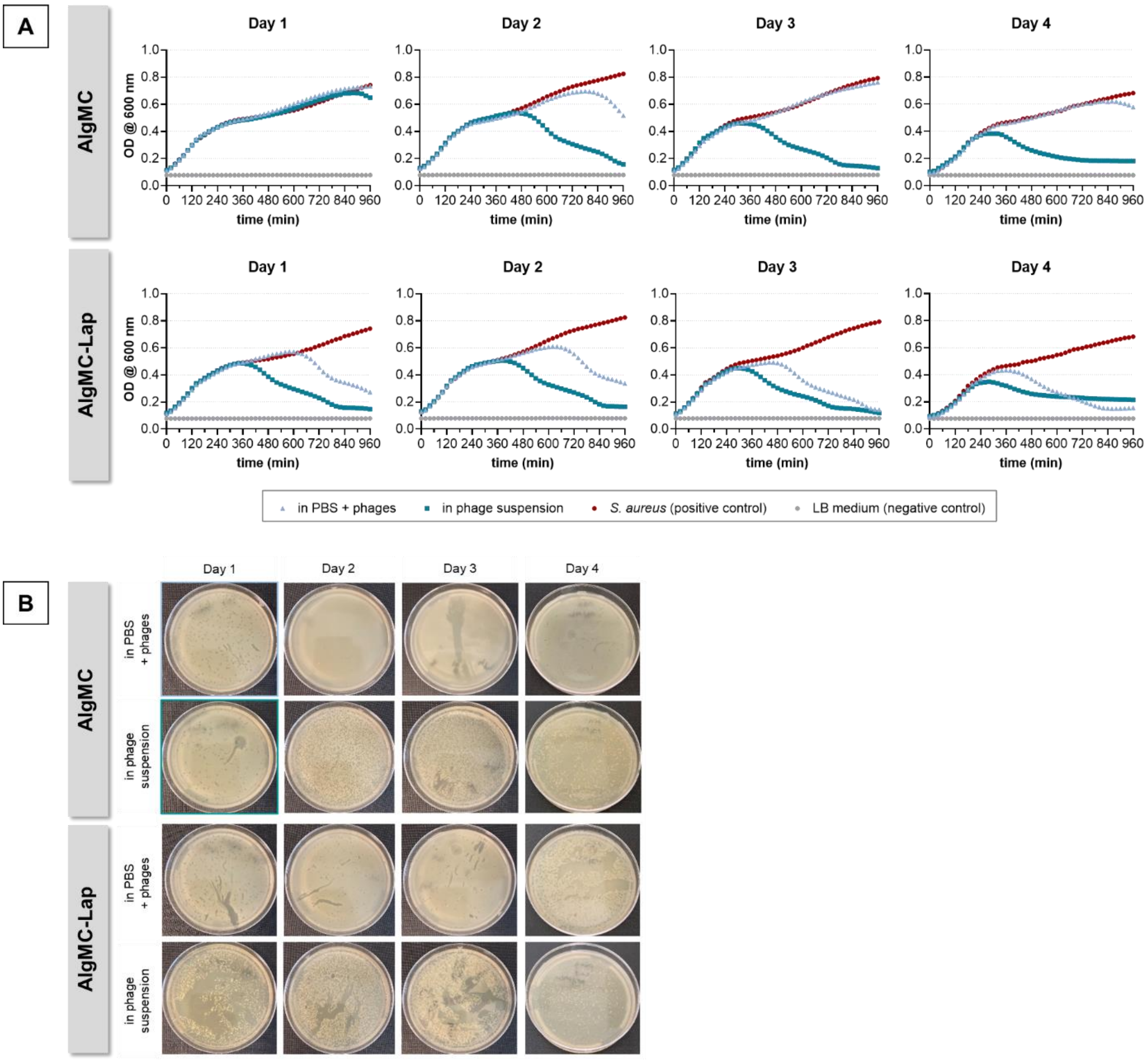

Supplemental figure S1. Antibacterial activity of phages released from printed AlgMC and AlgMC-Lap hydrogels. Phages were loaded into the inks either by mixing the phage suspension 1:10 with the ink prepared in PBS, yielding a concentration of approx. 2×10^{8} PFU mL^{-1} ('*in PBS + phages*') or by direct preparation of the ink in phage suspension, yielding a concentration of approx. 2×10^{9} PFU mL^{-1} ('*in phage suspension*'). Release was examined by incubating the samples in water at 37 °C for 4 days; every day an aliquot was taken for analysis without refreshing of the eluent: **(A)** *S. aureus* growth curve analysis; **(B)** plaque forming assay: 100 µL of the release solution was mixed with 1 mL *S. aureus* suspension (OD_{600} = 1) in 5 mL semi solid LB agar and poured

on the solid LB agar plates, followed by incubation at 37 °C for 16 h. Transparent areas/spots on the agar plates indicate active phages lysing the bacteria immobilised in the semi solid agar.

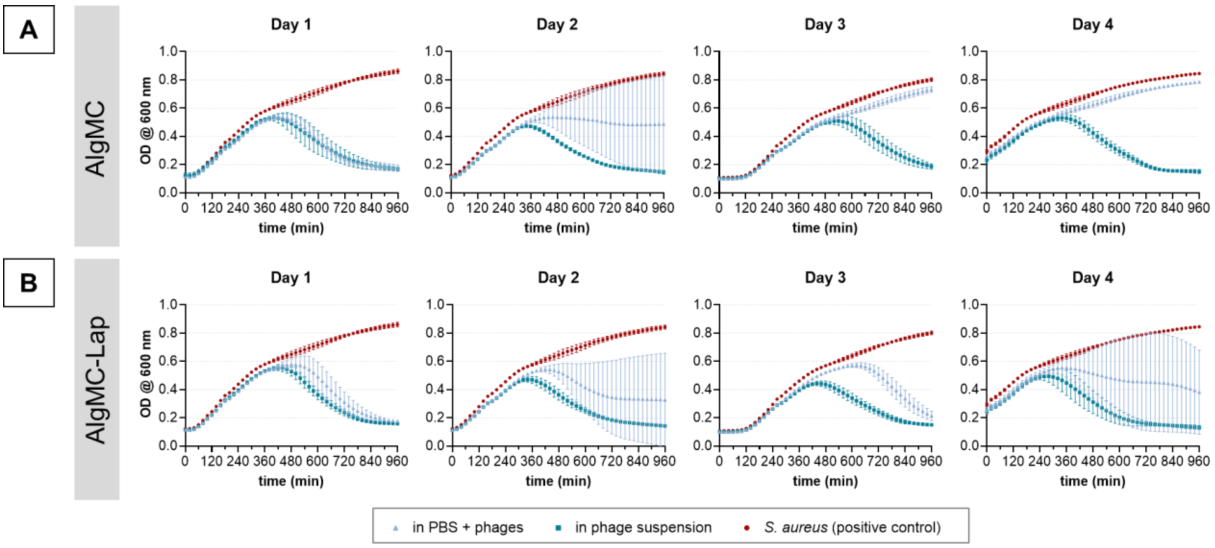

Supplemental figure S2. Antibacterial activity of phages released from printed **(A)** AlgMC and **(B)** AlgMC-Lap hydrogels: growth curve analysis without refreshing of the eluent. Phages were loaded into the inks either by mixing the phage suspension 1:10 with the ink prepared in PBS, yielding a concentration of approx. 2×10^9 PFU mL^{-1} (‘*in PBS + phages*’) or by direct preparation of the ink in phage suspension, yielding a concentration of approx. 2×1010 PFU mL^{-1} (‘*in phage suspension*’). Release was examined by incubating the samples in water at 37 °C for 4 days; every day an aliquot was taken for analysis (mean ± SD; n = 4).

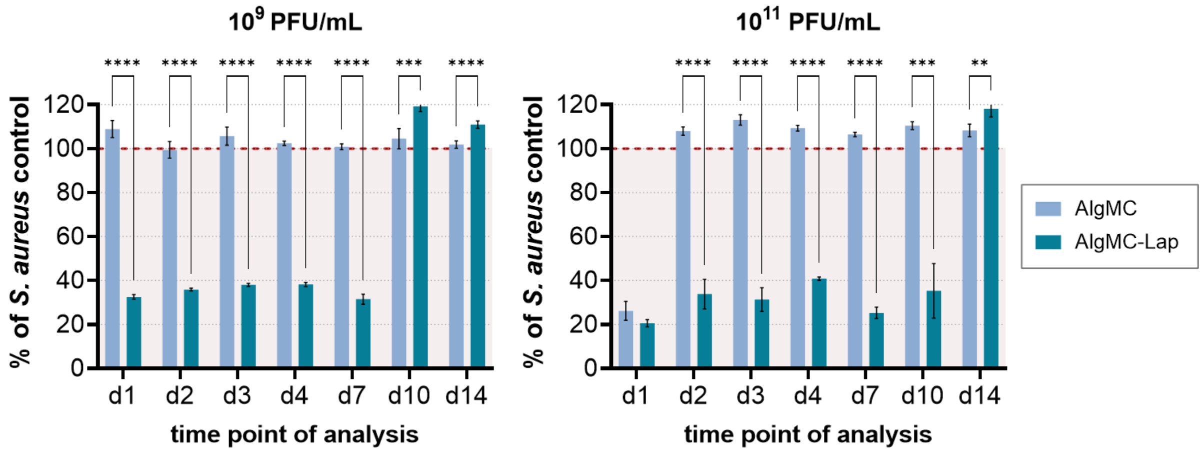

Supplemental figure S3. Antibacterial activity of phages released from printed AlgMC and AlgMC-Lap hydrogels: growth curve analysis with change of the eluent at each time point of analysis. The inks were prepared by dissolving the biomaterials in phage suspensions of either 1×10^9 or 1×10^{11} PFU mL^{-1}. Release was examined by incubating the samples in water at 37 °C. Growth curve end point measurement (16 h) for the release solutions related to the positive control with water (mean ± SD; n = 5; **** $p < 0.0001$, *** $p < 0.001$, ** $p < 0.01$).

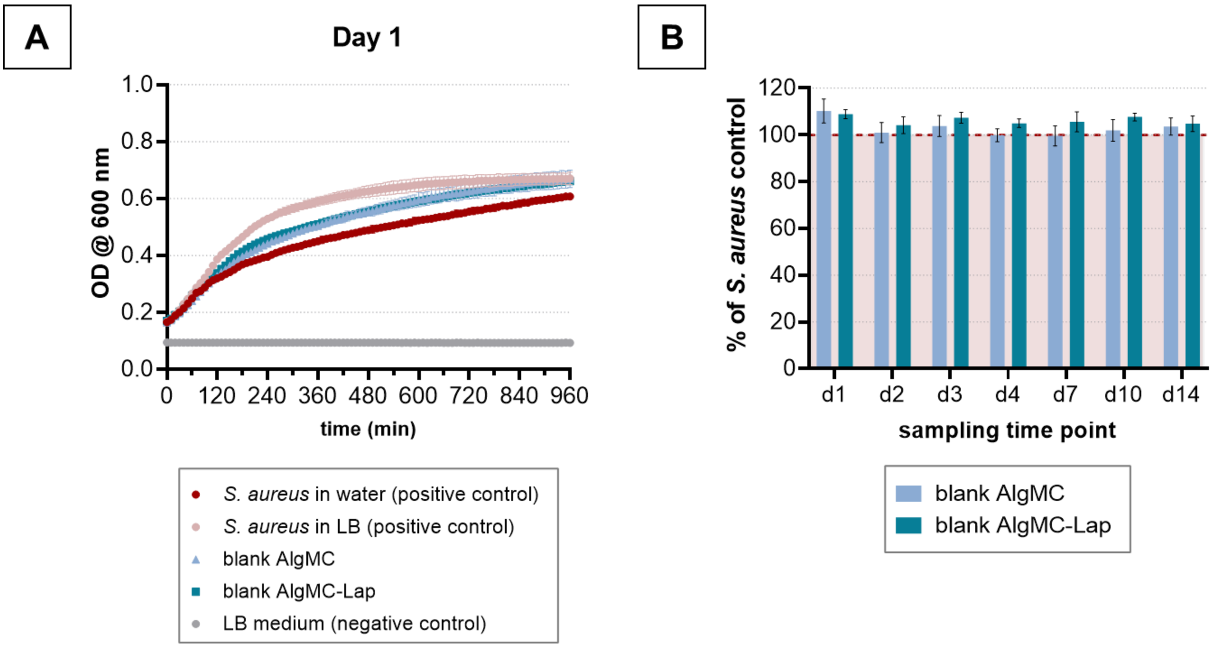

Supplemental figure S4. Antibacterial activity of phage-free (blank) printed AlgMC and AlgMC-Lap hydrogels incubated in water at 37 °C: **(A)** Growth curve at day 1 and **(B)** growth curve end point measurement (16 h) for the release solutions related to the positive control with water with regular refreshing of the eluent (mean ± SD; n = 5).

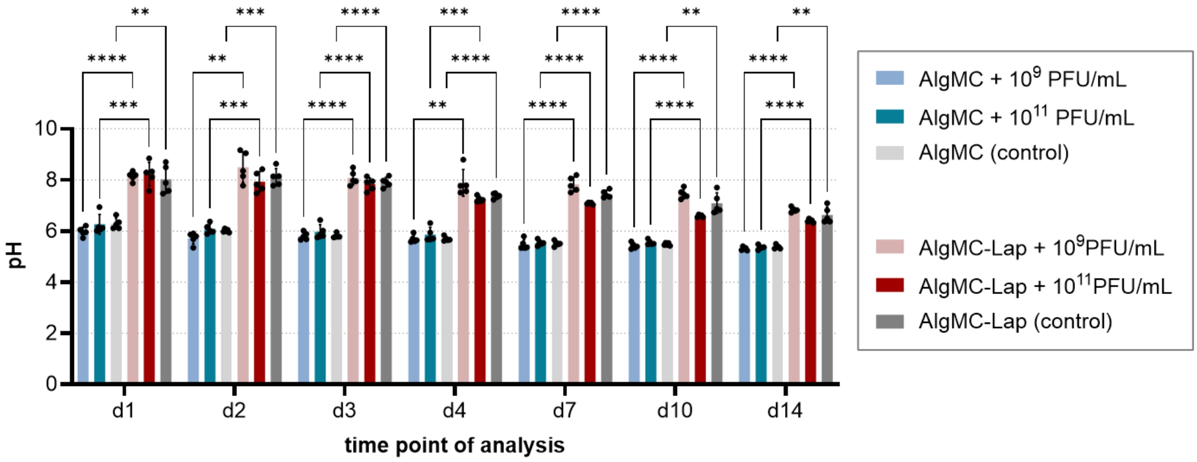

Supplemental figure S5. Measurement of the pH value in release solutions collected from printed AlgMC and AlgMC-Lap hydrogels (change of the eluent at each time point of analysis). The inks were prepared by dissolving the biomaterials in phage suspensions of either $1{\times}10^9$ or $1{\times}10^{11}$ PFU mL^{-1} or in TSB medium (control). Samples were incubated in water at 37 °C (mean ± SD; n = 5, **** $p < 0.0001$, *** $p < 0.001$, ** $p < 0.01$).

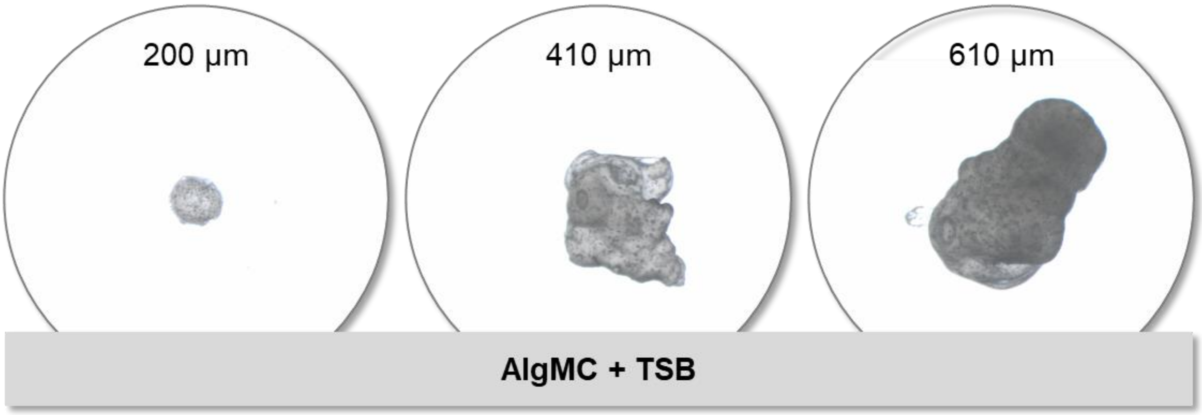

Supplemental figure S6. Printing 3D constructs from AlgMC inks. Assessment of mass flow using different needle diameters. The inks were prepared by dissolving the alginate and methylcellulose in phage-free TSB medium.

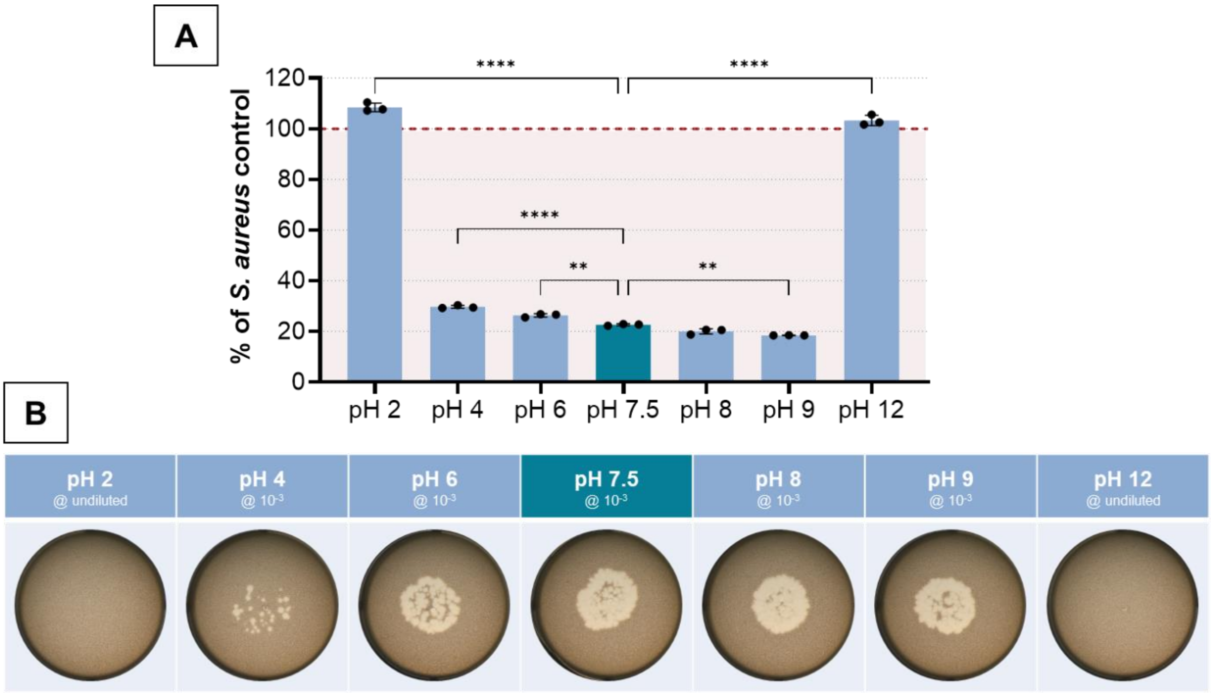

Supplemental figure S7. Influence of pH on phage stability. **(A)** Growth curve end point measurement (12 h; mean ± SD, n = 3, **** $p < 0.0001$, ** $p < 0.01$, One-Way ANOVA followed by Dunnett´s multiple comparison tes compared to pH 7.5) and **(B)** spot plaque assay. SM-phage buffer was adjusted to pH values ranging from 2-12 with HCl and NaOH, respectively. Then, 900 µL SM-buffer at the desired pH was mixed with 100 µL phage solution at 1×10^9 PFU/mL resulting in a final phage concentration of 1×10^8 PFU/mL. For controls, phage solution was replaced by SM-buffer. Samples were then incubated at 37 °C for 22 h following analysis via growth curve measurement and spot plaque assay. For better visualization of plaques, for the spot plaque assay undiluted phage suspensions were used for pH 2 and pH 12 (*'@ undiluted'*) whereas 1:1000 (*'@ 10^{-3}'*) diluted phage suspensions were used for pH 6-9.
